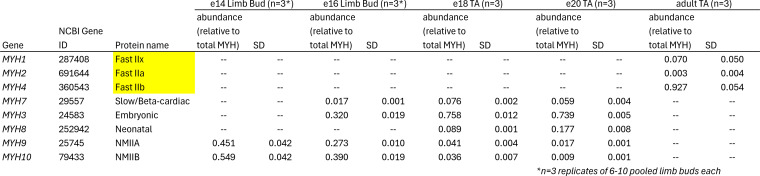# Correction: Functional role of myosin-binding protein H in thick filaments of developing vertebrate fast-twitch skeletal muscle

**DOI:** 10.1085/jgp.20241360411072024c

**Published:** 2024-11-13

**Authors:** Andrew F. Mead, Neil B. Wood, Shane R. Nelson, Bradley M. Palmer, Lin Yang, Samantha Beck Previs, Angela Ploysangngam, Guy G. Kennedy, Jennifer F. McAdow, Sarah M. Tremble, Marcus A. Zimmermann, Marilyn J. Cipolla, Alicia M. Ebert, Aaron N. Johnson, Christina A. Gurnett, Michael J. Previs, David M. Warshaw

Vol. 156, No. 12 | https://doi.org/10.1085/jgp.202413604 | October 07, 2024

The authors regret that, in the original article, the myosin isoform names IIx, IIa, and IIb were transposed in Figure 1 C and Table S1. The corrected figure and table appear here, and the figure legend remains unchanged. The corrections in Table S1 are highlighted.

Additionally, in the second paragraph of the Results section, “IIx” was mistakenly used instead of “IIb.” The corrected text is in bold: “Unique peptides were detected from fast-type myosin heavy chain isoforms IIa (MYH2), IIb (MYH4), and IIx (MYH1) (Table S1), with those from myosin heavy chain **IIb** being the most abundant (Fig. 1 C).”

The conclusions of the paper are not affected by these errors. The errors appear in PDFs downloaded before November 8, 2024.

**Figure 1.**
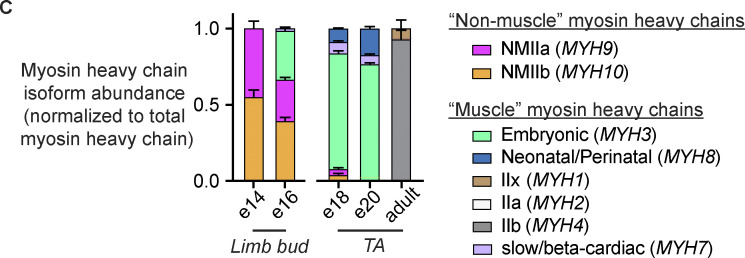
The sarcomere “C-zone” is home to the MyBP-C/H family of regulatory proteins.

**Table S1.**